# Case Report: REM sleep without atonia in an adult with pediatric acute-onset neuropsychiatric syndrome: a case study and mechanistic insights

**DOI:** 10.3389/frsle.2025.1654119

**Published:** 2025-11-19

**Authors:** Martina Mulas, Nazanin Biabani, Sean Higgins, Joshua Benson, Nikita Gurbani, Ana Santic, Danielle Wasserman, Valentina Gnoni, Karolina Poplewska, Katarina Ilic, Philip R. Holland, Panagis Drakatos, Alexander D. Nesbitt, David O'Regan, Monica Puligheddu, Ivana Rosenzweig

**Affiliations:** 1 Department of Medical Sciences and Public Health, Sleep Disorder Centre, University of Cagliari, Cagliari, Italy; 2 Department of Neuroimaging, Sleep and Brain Plasticity Centre, Institute of Psychiatry, Psychology and Neuroscience (IoPPN), King's College London (KCL), London, United Kingdom; 3 Sleep Disorders Centre, Guy's and St Thomas' NHS Foundation Trust, London, United Kingdom; 4 Department for Sleep Disorders, Psychiatric Clinic Vrapce, Zagreb, Croatia; 5 Department of Neurology and Sleep Center, Shaare Zedek Medical Center, Jerusalem, Israel; 6 Department of Clinical Research in Neurology, Centre for Neurodegenerative Diseases and the Aging Brain, University of Bari ‘Aldo Moro', Tricase, Italy; 7 Department of Neurology, Poznan University of Medical Sciences, Poznan, Poland; 8 Parkinson's Foundation Centre of Excellence, King's College Hospital, London, United Kingdom; 9 Department of Neuroimaging, BRAIN Centre, Institute of Psychiatry Psychology and Neuroscience, King's College London, London, United Kingdom; 10 Headache Group, Wolfson Sensory, Pain and Regeneration Centre, Institute of Psychiatry, Psychology and Neuroscience, King's College London, London, United Kingdom; 11 King's College London Centre for Human and Applied Physiological Sciences, London, United Kingdom; 12 Department of Neurology, Guy's and St Thomas' NHS Foundation Trust, London, United Kingdom; 13 Faculty of Life Science and Medicine, School of Basic and Medical Biosciences, King's College London, London, United Kingdom

**Keywords:** pediatric acute-onset neuropsychiatric syndrome, PANS, REM sleep without atonia, RSWA, neuroinflammation, basal ganglia, orexin, polysomnography

## Abstract

**Background:**

Pediatric Acute-Onset Neuropsychiatric Syndrome (PANS) is an immune-mediated disorder marked by abrupt onset of obsessive-compulsive symptoms and a spectrum of neuropsychiatric and somatic features, including sleep disturbances. Although polysomnographic studies increasingly document REM Sleep Without Atonia (RSWA) in children with PANS, persistence of RSWA into adulthood remains unreported and poorly understood.

**Case presentation:**

We report a 20-year-old woman with a 5-year history of relapsing-remitting neuropsychiatric symptoms consistent with PANS, including obsessive-compulsive features, complex tics, anxiety, and sleep disruption. The onset was temporally associated with a viral illness and followed by recurrent exacerbations triggered by infections and psychosocial stressors. Polysomnography, conducted during an inter-episode baseline, revealed RSWA with reduced REM atonia and fragmented sleep architecture, despite the absence of REM sleep behavior disorder (RBD). At onset, clinical findings included motor incoordination and sensorimotor hypersensitivities. Past serological workup supported a post-infectious inflammatory phenotype.

**Discussion:**

This case expands current understanding of PANS by documenting persistent RSWA in an adult patient, suggesting chronic disruption of REM-regulating neurocircuits. Mechanistically, we explore how basal ganglia autoimmunity, dopaminergic dysregulation, and hypothalamic orexin imbalance may converge to impair REM atonia. Emerging literature is consistent with RSWA as a state or trait marker of central neuroinflammation in neuroimmune conditions such as PANS. These findings underscore the diagnostic and pathophysiological relevance of sleep phenotyping in neuroinflammatory syndromes and call for longitudinal evaluation of sleep physiology across the disease course.

**Conclusion:**

RSWA may represent an under-recognized manifestation of chronic neuroimmune dysfunction in PANS. Its persistence into adulthood suggests long-term dysregulation of REM sleep circuitry and invites further investigation into the role of orexin and basal ganglia-mediated inhibition in neuroimmune disorders.

## 1 Introduction

Pediatric Acute-onset Neuropsychiatric Syndrome (PANS) and Pediatric Autoimmune Neuropsychiatric Disorder Associated with Streptococcal infections (PANDAS) are immunologically mediated conditions characterized by abrupt-onset obsessive-compulsive behaviors and a range of complex neuropsychiatric symptoms (Swedo et al., 2012; Frankovich et al., 2015; Vreeland et al., 2023). First described by Swedo et al. (2012), these disorders are increasingly recognized as post-infectious syndromes potentially involving autoantibodies against basal ganglia structures (Swedo et al., 2012; Frankovich et al., 2015; Vreeland et al., 2023). PANDAS, in particular, is classically triggered by Group A Streptococcal (GAS) infection, while PANS encompasses a broader array of potential triggers including viral infections, neuroinflammation, metabolic dysregulation, and endocrine perturbations (Lojek and Rzeszutek, 2025).

Clinically, PANS is defined by the sudden onset of obsessive-compulsive symptoms or restrictive eating accompanied by at least two additional neuropsychiatric features. These often include anxiety, tics, mood instability, irritability, aggression, sensory abnormalities, and cognitive regression (Swedo et al., 2012; Frankovich et al., 2015). Importantly, somatic symptoms such as enuresis, mydriasis, and notably, sleep disturbances, are also common (Swedo et al., 2012; Frankovich et al., 2015; Vreeland et al., 2023). Despite this, objective investigations of sleep in PANS remain scarce, with a dearth of data using polysomnography (PSG; also see Table 1).

**Table 1:** Summary of studies investigating sleep disturbances in PANS/PANDAS.

**References**	**Sample characteristics**	**Sleep assessment**	**Key findings**
Gaughan et al. (2016)	*N* = 15; *F* = 8; Mean age 7.2 years	Overnight PSG	87% had ≥1 abnormality; 53% exhibited REM motor disinhibition; 27% met RBD criteria; 33% had elevated PLMI; only 13% had normal PSG
Santoro et al. (2018)	*N* = 9; *F* = 2; Mean age 8 years	Overnight PSG	78% had elevated PLMI during REM (median 6.9/h); RSWA identified in 2 subjects by manual scoring
Gagliano et al. (2021)	*N* = 23; *F* = 16; Mean age 9.8 years	Overnight PSG	74% had ≥1 PSG abnormality; 65% RSWA; 59% sleep fragmentation; 47% PLMD; 78% reported snoring; 33% had obstructive sleep apnea (OSAS)
Congiu et al. (2024)	*N* = 69 PANS (*F* = 16, Mean age = 8.3 ± 3.1 years); *N* = 44 matched controls (*F* = 15, Mean age = 8.0 ± 3.1 years)	PSG and REM Atonia Index (RAI)	RAI significantly lower in PANS vs. controls; 25/69 patients had pathological RAI vs. 1/44 controls; PLM index higher in PANS
Congiu et al. (2024)	Single case report: Male child with PANS and later narcolepsy type 1	Clinical and sleep diagnostic assessment	Suggests potential shared autoimmune basis between PANS and narcolepsy type 1

This table summarizes representative studies evaluating sleep architecture and REM sleep abnormalities in children diagnosed with Pediatric Acute-onset Neuropsychiatric Syndrome (PANS) and Pediatric Autoimmune Neuropsychiatric Disorders Associated with Streptococcal infections (PANDAS). Data include sample characteristics, objective sleep assessments (primarily overnight polysomnography), and principal findings related to REM Sleep Without Atonia (RSWA), REM Behavior Disorder (RBD), Periodic Limb Movement Index (PLMI), and other features of disordered sleep continuity and architecture. F, the number of female participants; PLMI, Periodic Limb Movement Index; PLMS, Periodic Limb Movements of Sleep; PLM, Periodic Limb Movements; PSG, overnight Polysomnography; RAI, the REM Atonia Index; RBD, REM Behavior Disorder; REM, Rapid Eye Movement sleep; RSWA, REM Sleep Without Atonia; OSAS, Obstructive Sleep Apnea Syndrome; NT, Narcolepsy Type 1; HC, Healthy Controls used for comparative analyses.

Emerging literature has highlighted sleep abnormalities as a core, yet under-investigated, feature of PANS. Chang et al. (2015) and Swedo et al. (2012) reported that up to 84% of PANS patients experience sleep-related complaints including insomnia, parasomnias, nightmares, and sleep fragmentation. Recent studies using PSG have identified specific disturbances in REM sleep, particularly REM sleep without atonia (RSWA) and REM sleep behavior disorder (RBD), suggesting a potential dysfunction in brainstem and subcortical REM regulatory circuits (Table 1).

Gaughan et al. (2016) conducted one of the earliest PSG-based studies in this population and reported that 87% of children with active PANS symptoms had at least one REM sleep abnormality, including 27% who met full RBD criteria and 53% who exhibited REM motor disinhibition. These findings were corroborated by Santoro et al. (2018), who found elevated periodic limb movement indices (PLMI) in 78% of their PANS sample and identified RSWA in several patients upon manual scoring. Similarly, Gagliano et al. (2021) reported that 74% of PANS patients displayed at least one PSG abnormality, with 65% exhibiting RSWA and 47% meeting criteria for periodic limb movement disorder (PLMS). These studies collectively demonstrate that REM-related motor disinhibition could be considered a recurrent and quantifiable feature in PANS.

A more recent investigation Congiu et al. (2024) further substantiated these findings using the REM Atonia Index (RAI), a validated PSG metric for quantifying RSWA. In a sample of 69 children with PANS and 44 age- and sex-matched controls, the RAI was found significantly lower in the PANS group, with 25 PANS subjects showing pathological REM atonia levels compared to only one control, perhaps again in support of the potential of RSWA as a biomarker of central neuroinflammatory involvement in PANS.

**Figure 1:**
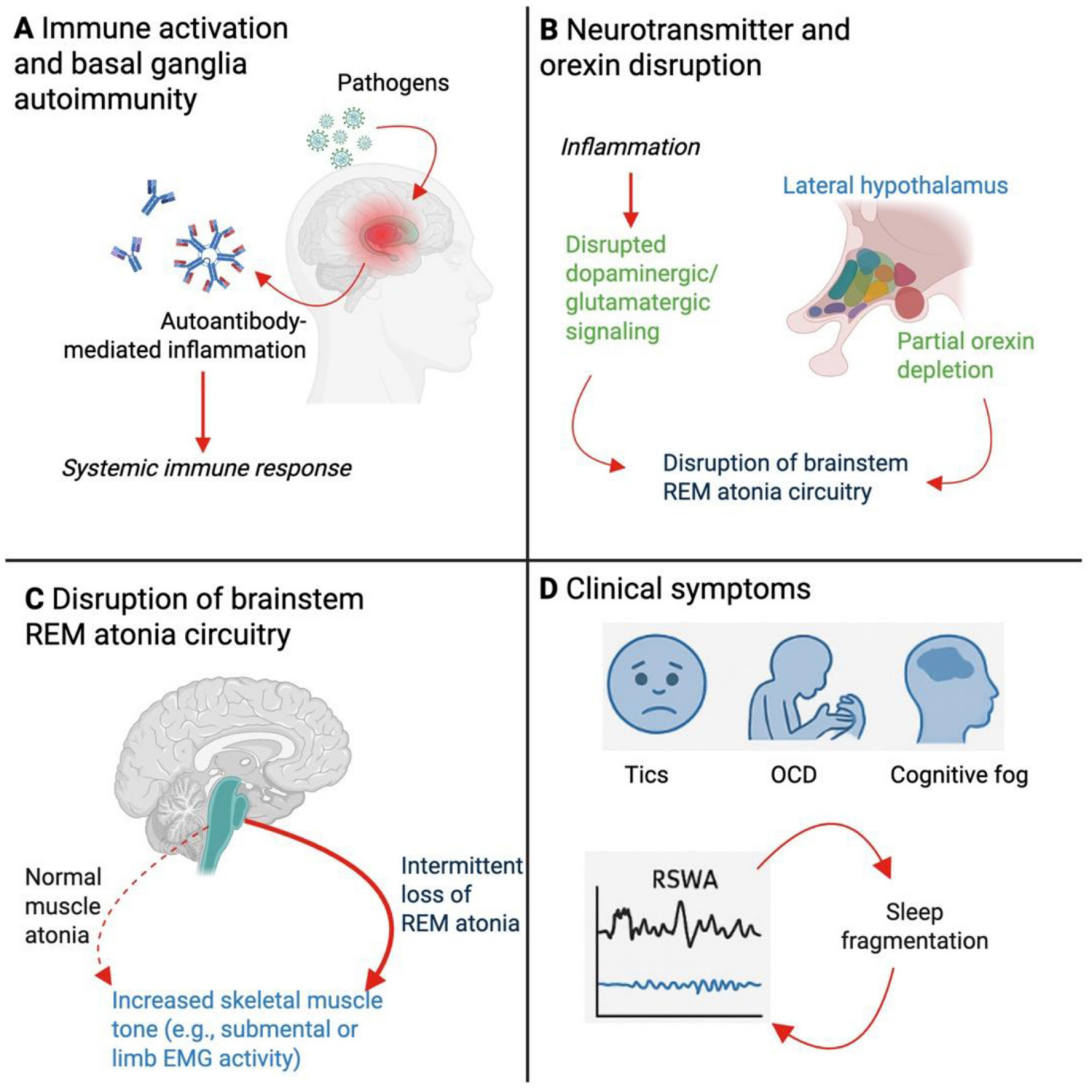
Mechanistic model linking neuroinflammation to REM Sleep Without Atonia (RSWA) in Pediatric Acute-Onset Neuropsychiatric Syndrome (PANS). This schematic illustrates the proposed pathophysiological cascade by which neuroinflammation in PANS contributes to disrupted REM sleep regulation and RSWA. **(A)** Systemic immune activation following infectious triggers (e.g. streptococcal or viral pathogens) initiates an autoimmune response, characterized by autoantibody production and neuroinflammation targeting the basal ganglia. This neuroinflammatory state may be sustained or episodic, contributing to chronic symptom fluctuations. **(B)** Inflammatory signaling disrupts neurotransmitter pathways, particularly dopaminergic and glutamatergic circuits projecting from the basal ganglia and hypothalamus. Partial depletion or dysregulation of orexin-producing neurons in the lateral hypothalamus contributes to destabilization of sleep-wake regulation. These changes impair normal signaling to pontine nuclei responsible for generating muscle atonia during REM sleep. **(C)** Dysfunctional integration of REM atonia circuitry in the brainstem, including structures such as the sublaterodorsal tegmental nucleus and medullary reticular formation, results in failure to inhibit skeletal muscle tone during REM sleep. This manifests as persistent REM-associated muscle activity, quantified as RSWA on polysomnography. The resulting disruption in normal motor inhibition is conceptualized here as a failure to suppress tone in representative skeletal muscle groups. **(D)** Clinically, RSWA contributes to fragmented REM sleep and altered sleep architecture, further exacerbating neuropsychiatric symptoms including complex tics, obsessive-compulsive behaviors (OCD), and cognitive dysfunction. Sleep fragmentation may, in turn, amplify neuroinflammatory processes and perpetuate symptom cycles.

Notably, a case report by Congiu et al. (2024) described a child with concurrent narcolepsy type 1 (NT1) and PANS, arguing for a shared immunological spectrum. Wenz et al. (2023) similarly described three children diagnosed with both NT1 and Sydenham's chorea, reinforcing the hypothesis that post-infectious autoimmune disorders may converge upon common neurobiological pathways affecting REM regulation (Figure 1).

Traditionally, several models have been proposed to explain REM sleep abnormalities in PANS. Neuroimaging studies and serological findings consistently demonstrate basal ganglia involvement, including autoantibody targeting of dopamine D1 and D2 receptors, tubulin, and lysoganglioside GM1 (Frankovich et al., 2015; Vreeland et al., 2023). These autoantibodies may interfere with cortico-striato-thalamo-cortical loops and disrupt projections to brainstem nuclei responsible for REM atonia (Figure 1). Furthermore, basal ganglia and thalamic inflammation may alter downstream activity in the sublaterodorsal tegmental nucleus and medullary inhibitory centers that regulate muscle tone during REM sleep (Figure 1).

Dopaminergic dysfunction is another likely contributor. Dopamine modulates motor inhibition and arousal, and its dysregulation is associated with both RBD and PLMS (Drakatos et al., 2021; Wasserman et al., 2021). Inflammatory insult to dopaminergic circuits in PANS may, thus, arguably, simultaneously act to produce tics, executive dysfunction, and REM disinhibition. Supporting this view, iron deficiency, a common metabolic finding in PANS, impairs tyrosine hydroxylase activity, reducing dopamine synthesis and compounding sleep-related motor instability (Drakatos et al., 2021; Daubner et al., 2011).

Another putative player in REM modulation could be the orexin (hypocretin) system. Orexin neurons, located in the lateral hypothalamus, orchestrate arousal, REM transitions, and maintenance of muscle atonia through projections to the brainstem (Concetti and Burdakov, 2021). Orexin deficiency is the hallmark of NT1, and partial orexin dysregulation may result in fragmented REM and RSWA without full narcoleptic features (Antelmi et al., 2020). Emerging evidence from preclinical studies suggests that chronic inflammation may activate a reserve population of orexin-expressing neurons in a compensatory, yet dysregulated, manner, leading to hyperarousal and REM sleep instability (James and Aston-Jones, 2022; Clark and Vissel, 2014). This concept may be particularly relevant to PANS, where immune dysregulation, stress, and fluctuating orexin tone may underlie overlapping features with NT1.

Importantly, while RSWA (when accompanied with dream re-enactment) is well-recognized as a harbinger of neurodegeneration in synucleinopathies (Högl et al., 2018), its presence in pediatric autoimmune conditions such as PANS may reflect a reversible, functional disturbance. Observational studies have shown that RSWA and related PSG abnormalities may improve with immunotherapy, including corticosteroids and intravenous immunoglobulin (IVIG), suggesting that sleep phenotyping could serve as a treatment-responsive biomarker.

In light of these findings, we undertook a critical literature synthesis on sleep disturbances in PANS and presents a novel adult case of persistent RSWA (see Table 1). To our knowledge, this is the first documented case of an adult with longstanding PANS exhibiting polysomnographically confirmed RSWA. We propose a mechanistic model in which basal ganglia autoimmunity, dopaminergic and orexinergic dysregulation, and inflammatory stress converge to impair REM motor control. By extending our understanding of REM pathology beyond degenerative contexts, we advocate for the integration of sleep evaluation into the diagnostic framework for PANS and related neuroimmune syndromes.

## 2 Methodology and case presentations

### 2.1 Methods

A narrative literature review with a targeted focus on sleep disturbances in Pediatric Acute-onset Neuropsychiatric Syndrome (PANS) and Pediatric Autoimmune Neuropsychiatric Disorders Associated with Streptococcal Infections (PANDAS) was conducted. The objective was to synthesize clinically and mechanistically relevant findings, including polysomnographic (PSG) observations, sleep architecture disruptions, and underlying neuroimmunological mechanisms, to contextualize a representative clinical case.

A systematic literature search was performed in February 2025 across three major biomedical databases: MEDLINE (via PubMed), Embase, and Scopus. The search strategy combined controlled vocabulary and keyword terms using Boolean operators as follows:

(“Pediatric Acute-onset Neuropsychiatric Syndrome” OR “PANDAS” OR “PANS” OR “Pediatric Autoimmune Neuropsychiatric Disorder associated with streptococcal infection” OR “obsessive-compulsive disorder” OR “OCD”) AND [“sleep disturbances” OR “sleep disorders” OR “sleep” OR “insomnia” (MeSH Terms)].

The search was limited to full-text, peer-reviewed articles published in English. Eligible sources included original research articles (e.g., cohort, case-control, cross-sectional studies), case reports, and systematic or narrative reviews. Titles and abstracts were screened manually, and full-texts were reviewed for relevance and methodological rigor.

Studies were included if they met the following criteria: involved children or adolescents diagnosed with PANS or PANDAS (or equivalent clinical features consistent with current definitions); reported sleep-related findings derived from objective assessments (e.g., PSG, sleep diaries) or structured clinical observations; included sample details and outcomes relevant to sleep architecture, parasomnias, REM physiology, or associated neuropsychiatric symptomatology.

A total of five articles met all inclusion criteria and were included in this review. These studies were subsequently summarized in tabular form (Table 1), detailing sample characteristics, sleep measures used, and key findings. A sixth source, a case report involving comorbid PANS and narcolepsy type 1, was included due to its mechanistic relevance to REM sleep dysregulation and autoimmune overlap.

#### 2.1.1 Polysomnography acquisition and RSWA/RAI quantification

Overnight video-polysomnography (vPSG) was performed with a standard adult montage, as previously published (Wasserman et al., 2022; Troester et al., 2023). REM epochs were identified from scorer annotations and segmented into 30-s epochs and 3-s mini-epochs for EMG analysis (Troester et al., 2023). Submentalis EMG was high-pass filtered at 10 Hz, low-pass at 100 Hz, with 50/60 Hz notch, full-wave rectified and smoothed with a 100-ms moving average. From the three chin derivations, the lowest-artifact channel was used consistently for all metrics. RSWA metrics (AASM/SINBAR-compatible; Ferri et al., 2010; McCarter et al., 2017; Frauscher et al., 2012; Medicine, 2023): tonic RSWA was defined as ≥50% of a 30-s REM epoch above 2 × REM baseline amplitude; phasic bursts were 0.1–5.0 s events at ≥2 × baseline amplitude. Phasic % was the proportion of 3-s mini-epochs containing ≥1 burst, reported for chin and tibialis. REM Atonia Index (RAI) was computed on 3-s mini-epochs adapting previous classification (atonia/intermediate/non-atonia) by Ferri et al. (2010) and calculated as: RAI = (N_atonia + 0.5 × N_intermediate) / N_total (range 0–1; lower values indicate less atonia). Phasic chin EMG exceeded McCarter/Frauscher thresholds for iRBD-level RSWA; tonic fell below the 30% threshold; RAI was in the abnormal range (McCarter et al., 2017; Frauscher et al., 2012). Where artifact precluded reliable analysis, affected REM segments were excluded *a priori*.

### 2.2 Case presentation

We report the case of a 20-year-old woman with a 5-year history of relapsing-remitting neuropsychiatric symptoms, initially precipitated by a post-infectious immune episode, with features consistent with PANS. The case is notable for confirmed REM Sleep Without Atonia (RSWA) on polysomnography and a complex constellation of motor, cognitive, and affective abnormalities with prominent neurologic features.

#### 2.2.1 Premorbid functioning and initial presentation

The patient demonstrated exceptional neurodevelopmental and academic functioning until mid-adolescence. At age 15, she experienced an abrupt and dramatic onset of obsessive-compulsive behaviors, complex tics, and emotional lability following a documented viral illness. The initial episode occurred in the context of significant psychosocial stress and was marked by regressive behaviors, speech dysfluency, sensory hypersensitivity, and behavioral disorganization.

Symptoms included rapid onset of involuntary motor movements, including facial and cervical tics, stereotypic limb jerks, and whole-body startle-like phenomena. These were accompanied by intrusive vocalizations and complex verbal perseveration. The patient exhibited episodic behavioral regression with abrupt shifts into childlike affect, altered gait, transient mutism, and emotionally labile states.

#### 2.2.2 Course and exacerbations

The illness progressed into a relapsing-remitting pattern, with symptom exacerbations temporally associated with febrile illnesses, upper respiratory infections, and significant psychological stressors. Inter-episode periods were marked by residual obsessive-compulsive traits, anxiety, and cognitive inefficiency.

Notably, symptom relapses were often heralded by a cluster of neurologic and behavioral signs: resurgence of complex tics, sensorimotor hypersensitivities, motor incoordination, attentional dysregulation, and episodes of dissociation. Several episodes included impaired gait and transient dysarthria. During exacerbations, involuntary movements became more intrusive and difficult to suppress, consistent with a complex tic disorder phenotype. No epileptiform activity was documented, but one episode involved stereotyped eye-rolling and unresponsiveness, prompting consideration of a non-epileptic event.

#### 2.2.3 Cognitive and academic profile

Premorbidly, the patient exhibited high cognitive aptitude with advanced reading comprehension and verbal recall. Since illness onset, she has reported persistent difficulties with executive functioning, sustained attention, and memory retrieval, which have significantly impaired her academic trajectory. Though currently enrolled in a university programme, she reports marked challenges with academic work.

Neuropsychiatric features have included pervasive anxiety, particularly separation anxiety, mood lability, episodic rage attacks, obsessive-compulsive rituals centered on symmetry and sensory input, and intrusive ideation. Dissociative episodes during flares were notable for regression into childlike speech and affect. There were intermittent paranoid and self-referential thoughts during exacerbations, though no sustained psychosis was observed.

#### 2.2.4 Neurologic examination

In past, clinical examination during initial exacerbations revealed motor overflow phenomena, impaired rapid alternating movements, and subtle dysmetria. Gait during episodes was broad-based with impaired tandem walk. The patient exhibited heightened startle responses and deficits in fine motor coordination. These findings are consistent with cortico-striatal circuit dysfunction and resemble patterns seen in post-infectious basal ganglia syndromes.

#### 2.2.5 Sleep symptoms and polysomnography

The patient reported a longstanding history of disturbed sleep, characterized by excessive daytime sleepiness, prolonged nocturnal latency, vivid nightmares, and increased sleep fragmentation during symptomatic periods. Subjectively, she reported episodes of somnolence occurring in inappropriate contexts and unrefreshing sleep. These complaints persisted throughout the illness course but improved modestly during symptom remissions.

An inter-episode video-polysomnographic study (vPSG) demonstrated objective REM sleep without atonia (RSWA (McCarter et al., 2017; Frauscher et al., 2012); see Supplementary Video S1). Across all scored REM, tonic chin EMG was 23.0%, phasic chin EMG 36.7%, and phasic tibialis 21.7% (left 21.7%, right 17.4%); the REM Atonia Index (RAI; Ferri et al., 2010) was 0.45 (0–1; lower indicates less atonia). Sleep architecture showed REM latency of 88.0 min, arousal index of 20.5/h, apnea/hypopnea index of AHI 0.1 ev/h (RDI 0.1 ev/h); for other parameters please refer to Table 2. No REM sleep behavior disorder (RBD) was reported clinically, or observed on vPSG. At the time of PSG, the patient was not taking antidepressants, antipsychotics, benzodiazepines, or other agents known to modulate REM atonia. Following PSG, a multiple sleep latency investigation (MSLT; Supplementary Table S1) showed mean sleep latency of 16.5 min with only one sudden onset REM sleep period (SOREMP; REM latency 11 min in the single nap with sleep); ICSD-3 criteria for narcolepsy were not met (Medicine, 2023). Definitions and signal-processing steps for RSWA/RAI are detailed in Methods, and the full PSG metrics are summarized in Table 2.

**Table 2:** Overnight vPSG architecture, respiratory indices, and REM atonia metrics (RSWA/RAI).

**Metric**	**Value**
**Anthropometrics**	
BMI (kg·m^−2^)	24.2
**Sleep architecture**	
Total sleep time (TST), min	429.5
Sleep efficiency (SE), %	90.8
Sleep-onset latency (SOL), min	28.5
Wake after sleep onset (WASO), min	14.5
REM latency from sleep onset (REML), min	88.0
Stage distribution (% TST): N1 / N2 / N3 / REM	6.1 / 37.3 / 34.2 / 22.5
Arousal index, events·h^−1^	20.5
**Respiratory**	
AHI (3%/arousal rule), events·h^−1^	0.1
RDI, events·h^−1^	0.1
REM-AHI / Supine-AHI, events·h^−1^	0.0 / 0.5
Oxygen saturation (SpO_2_), mean / nadir, %	96.6 / 92.0
**Limb activity**	
PLMI, events·h^−1^	0.7
Isolated limb movement index, events·h^−1^	3.4
**REM atonia metrics (RSWA/RAI)**	
Tonic chin EMG, %	23.0%
Phasic chin EMG, %	36.7%
Phasic tibialis EMG, %	21.7% (L 21.7 / R 17.4)
REM Atonia Index (RAI), 0–1 (lower = less atonia)	0.45

Data from the baseline diagnostic vPSG. Architecture and respiratory indices include TST, SE, SOL, WASO, REML, stage distribution, Arousal Index, AHI/RDI (AASM 3%/arousal definition), REM-AHI, supine-AHI, and oxygenation. RSWA metrics were quantified from the lowest-artifact chin derivation using AASM/SINBAR-compatible rules (tonic and phasic percentages) and a Ferri-style REM Atonia Index (RAI). Values are reported as minutes, events·h^−1^, or percentages as indicated. AASM, American Academy of Sleep Medicine; AHI, Apnea-Hypopnea Index; AI, Arousal Index; EMG, Electromyography; PLMI, Periodic Limb Movement Index; PSG, Polysomnography; RAI, REM Atonia Index; RBD, REM sleep Behavior Disorder; RDI, Respiratory Disturbance Index; REM, Rapid Eye Movement sleep; REML, REM Latency; RSWA, REM Sleep Without Atonia; SE, Sleep Efficiency; SOL, Sleep-Onset Latency; SpO_2_, peripheral oxygen saturation; TST, Total Sleep Time; vPSG, video-Polysomnography; WASO, Wake After Sleep Onset.

Laboratory and Neuroimaging Investigations: Serological studies were done during initial pediatric presentation (see Supplementary Table S2 for the timeline) and elevated anti-streptolysin O (ASO) titres (>800 IU/mL) were reported, with a positive antinuclear antibody (ANA) at 1:160 in a speckled pattern. Ferritin levels were borderline low. Brain MRI, including high-resolution sequences of the basal ganglia and hypothalamus, was structurally unremarkable. Cerebrospinal fluid analysis and orexin quantification were not performed.

#### 2.2.6 Treatment history and response

At the pediatric onset of symptom flares, the patient received multiple empirical courses of antibiotics (amoxicillin-clavulanate). These were reported as temporally associated with partial mitigation of neuropsychiatric features, if administered early. Psychotropic agents trialed in past also included propranolol and low-dose Selective Serotonin Reuptake Inhibitors (SSRIs), with only modest symptomatic benefit. Cognitive behavioral therapy was associated with limited reductions in reactive aggression but did not prevent relapse. She has not received immunomodulatory therapies such as corticosteroids, intravenous immunoglobulin (IVIG), or plasmapheresis. Similarly, sleep-targeted pharmacologic interventions have not been initiated in past due to patient's reluctance and preference for non-pharmacological approaches.

#### 2.2.7 Current functioning and prognosis

At her presentation in the Sleep Disorders center, the patient complained of continuing to experience episodic neuropsychiatric deterioration approximately every 3–4 months. Between episodes, she retains partial academic functionality but reports persistent sensory intolerance, cognitive impairment, and fatigue. She remains socially dependent and has difficulty establishing age-appropriate peer relationships.

The persistence of RSWA on PSG during a baseline state suggests that REM motor dysregulation may represent a trait marker of underlying neuroimmune dysfunction rather than a transient symptom of acute exacerbation. The co-occurrence of complex tics, executive dysfunction, and sleep dysregulation points toward an integrative pathophysiology involving basal ganglia and hypothalamic circuits.

## 3 Discussion

This case offers novel insight into the neurobiological mechanisms underpinning REM sleep abnormalities in Pediatric Acute-onset Neuropsychiatric Syndrome (PANS), particularly REM Sleep Without Atonia (RSWA), by presenting the first known adult case with longstanding PANS exhibiting persistent RSWA on polysomnography. It raises key questions regarding the underlying pathophysiology of sleep regulation in PANS and provides a conceptual framework to understand how chronic neuroinflammation, basal ganglia dysfunction, and orexinergic dysregulation may intersect to produce motor disinhibition during REM sleep.

RSWA, characterized by abnormal muscle tone during REM sleep, is traditionally associated with REM Sleep Behavior Disorder (RBD) and is considered an early marker for neurodegenerative diseases, such as Parkinson's disease and dementia with Lewy bodies (Högl et al., 2018). However, in inflammatory or autoimmune contexts, RSWA may reflect transient or reversible dysfunction in REM atonia pathways. This is increasingly supported by observations in pediatric cohorts with PANS/PANDAS, where high rates of RSWA and RBD-like features have been detected without evidence of neurodegeneration (Gaughan et al., 2016; Santoro et al., 2018; Gagliano et al., 2021; Congiu et al., 2024).

The central hypothesis supported by this case is that immune-mediated neuroinflammation, particularly affecting the basal ganglia, thalamus, and brainstem REM regulatory nuclei, disrupts motor inhibition during REM sleep (Figure 1). Neuroimaging and serological studies in PANS frequently indicate inflammatory lesions in these regions, with a strong emphasis on basal ganglia involvement (Swedo et al., 2012; Frankovich et al., 2015). Autoantibodies targeting dopamine receptors (D1, D2), tubulin, and lysoganglioside-GM1 have been consistently identified in patients with PANS and Sydenham's chorea, another post-streptococcal autoimmune disorder (Vreeland et al., 2023). These antibodies may disrupt striatal-thalamocortical loops and interfere with inhibitory control of motor outputs during REM sleep.

As mentioned, dopaminergic dysfunction likely plays a pivotal role. The substantia nigra and ventral tegmental area (VTA), key dopaminergic hubs, project to both limbic and motor regions and modulate REM sleep via downstream connections to pontine reticular structures (Liu et al., 2020). Inflammatory interference with these circuits could explain co-occurring features such as tics, cognitive dysfunction, mood lability, and REM dysregulation (Figure 1). The frequent finding of periodic limb movements during REM (PLMS) in PANS further implicates dysregulated dopamine synthesis and signaling, as PLMS is well-known to respond to dopaminergic therapies and is often associated with iron deficiency (Gagliano et al., 2021; Drakatos et al., 2021).

Indeed, iron metabolism might be an underappreciated factor in the pathophysiology of REM abnormalities in PANS. Iron is a cofactor for tyrosine hydroxylase, the rate-limiting enzyme in dopamine synthesis (Daubner et al., 2011). Low ferritin levels have been repeatedly observed in PANS cohorts and, in conjunction with immune activation, may create a neurochemical milieu that is permissive for motor instability during REM (Santoro et al., 2018). This metabolic vulnerability may further destabilize cortico-striatal-thalamocortical loops and dopaminergic tone.

More recently, the possible role of the orexin system in PANS and sleep regulation has come into focus (Congiu et al., 2024; Knez et al., 2022). Orexin neurons, located in the lateral hypothalamus, are essential for maintaining wakefulness, regulating REM transitions, and enforcing REM muscle atonia via their projections to pontine inhibitory centers (Concetti and Burdakov, 2021). Loss of orexin-producing neurons is the defining feature of narcolepsy type 1 (NT1), which presents with RSWA, cataplexy, and disrupted REM control (Antelmi et al., 2020). Although CSF orexin levels were not measured in this case, the clinical overlap with NT1 raises the possibility of partial orexinergic dysregulation.

Preclinical studies have introduced the concept of an “orexin reserve,” a subpopulation of dormant or immature orexin-expressing neurons that can be recruited during high arousal, stress, or inflammation (James and Aston-Jones, 2022). Clark and Vissel (Clark and Vissel, 2014) suggest that under chronic neuroimmune stress, these reserve neurons may be pathologically activated, leading to a state of heightened arousal and impaired REM inhibition. In PANS, ongoing immune activation, cytokine release, and possible hypothalamic inflammation could activate these reserve circuits, producing hyperarousal, insomnia, and RSWA in the absence of complete orexin neuron loss (Figure 1).

This model is particularly relevant in light of the co-occurrence of PANS with narcolepsy and other sleep-related autoimmune disorders. Congiu et al. (2024) described a child with both NT1 and PANS, and Wenz et al. (2023) reported cases with overlapping NT1 and Sydenham's chorea. These findings support notion that post-infectious autoimmune conditions may affect shared sleep-regulating neural networks, particularly those involving orexin and dopamine.

In the reported case in our study, the patient has never received corticosteroids, intravenous immunoglobulin (IVIG), or other immunomodulatory treatments, however, existing literature suggests that REM sleep abnormalities in PANS may be responsive to such therapies (Swedo et al., 2012). Observational reports indicate that sleep parameters, including RSWA, may improve in tandem with clinical recovery following immune-targeted interventions (Frankovich et al., 2015). Thus, further evaluation of immunomodulatory approaches in this case may provide insights into the reversibility of REM dysfunction.

From a diagnostic standpoint, the presence of RSWA and REM fragmentation in PANS should prompt clinicians to consider a broader differential diagnosis that includes neuroimmune and sleep regulatory dysfunctions. Currently, sleep abnormalities in PANS are often under-recognized or misattributed to psychiatric comorbidities. We suggest that standardized PSG evaluation and REM Atonia Index (RAI) quantification could provide objective biomarkers of disease activity and treatment response.

In this particular case, the patient was reassured with findings, and at that present time, reluctant to engage in further treatment or follow up. A pragmatic plan in case of change of the clinical presentation was presented including a repeat vPSG within 3–6 months, with the same montage, and RSWA quantification, as objective outcome measures. Moreover, it was proposed to monitor iron status, and to consider corrections if ferritin < 75 μg/L, given dopamine/PLMS links. Furthermore, recommendation for potential additional behavioral sleep interventions were similarly considered (for instance sleep scheduling, stimulus control, or cognitive behavioral therapy for insomnia (CBT-I), if indicated). Clinically, immunomodulation should be considered by clinicians, if clinical and laboratory criteria support an inflammatory flare. Ideally, multi-disciplinary approach should be followed with sleep physicans documenting pre/post-treatment RSWA/RAI as response biomarkers.

Importantly, this case contributes to the growing body of evidence that PANS can persist into adulthood (Bodner et al., 2001; Deshmukh et al., 2022), challenging the notion that it is strictly a pediatric disorder. Presently due to the scarcity of the reports in the literature it is difficult to gauge any potential differences in pediatric vs. adult phenotype. As discussed, pediatric cohorts with PANS appear to include REM-related motor disinhibition on vPSG, including elevated RSWA/low RAI and REM-periodic limb movements (see Table 1). The present case demonstrates persistence of RSWA into early adulthood, despite otherwise normal respiratory indices and low PLMI, extending the pediatric observations into the adult spectrum. This supports the working hypothesis that in PANS, REM atonia circuitry could be chronically susceptible to immune-mediated dysregulation beyond childhood. In future it would be of interest to investigate adult patients presenting with treatment-resistant OCD, tics, and sleep disturbances, and to screen them for a history of acute-onset symptoms and infection-related relapses. Our patient, who continued to experience RSWA years after disease onset, exemplifies the need for longitudinal follow-up and continued research into the chronicity and progression of PANS.

There are several important limitations to this case, including the absence of CSF orexin measurement and neuroimaging markers specific to inflammation. However, the reported temporal association of immune flares with symptom recurrence, the documented PSG findings, and the immune-responsive pattern of symptom recurrence, provide strong, if circumstantial, evidence for an immune-mediated REM sleep phenotype. Future studies should integrate multimodal imaging, serum and CSF autoantibody profiling, and longitudinal PSG to better delineate the pathophysiology and treatment response in PANS.

In conclusion, this case supports a mechanistic model in which chronic neuroinflammation, basal ganglia-autoantibody interference, dopaminergic disruption, and orexinergic dysregulation coalesce to produce REM sleep disinhibition in PANS. RSWA in this context is not merely a parasomnia but may represent a key physiological marker of CNS inflammation. As such, sleep phenotyping should be incorporated into clinical evaluations and research frameworks for PANS and other neuroimmune disorders.

## Data Availability

The original contributions presented in the study are included in the article/Supplementary material, further inquiries can be directed to the corresponding author.

## Supplementary material

Supplementary Video S1 REM sleep without atonia in an adult with pediatric acute-onset neuropsychiatric syndrome (PANS). A fragment of vPSG materials shows elevated limb EMG activity followed by elevated submental EMG signal, demonstrating the characteristic loss of REM atonia. Channels are displayed from top to bottom: EOG (E1—M1 and E2—M2), EEG (F3—M2, F4—M1, C3—M2, C4—M1, O1—M2, O2—M1), ECG (ECG-LA—ECG-RA), submental EMG (CHIN1—CHINz, CHIN2—CHINz, CHIN1—CHIN2), bilateral anterior tibialis EMG (RLEG^+^–RLEG, LLEG^+^–LLEG), airflow (XFlow), snore and flow digital recording (Snore_DR and Flow_DR, respectively), thoracic and abdominal effort plethysmography (Chest and Abdomen, respectively), pulse oximetry (SpO_2_, %), and pulse rate (PR, bpm). This episode lasted approximately 13 s. bpm, beats per minute; EEG, electroencephalography; EOG, electrooculography; RBD, REM sleep behavior disorder; REM, rapid eye movement; vPSG, video polysomnography; %, percentage. Channels' codes are described above.

Supplementary Table S1 Multiple Sleep Latency Test (MSLT) findings at baseline assessment.

Supplementary Table S2 CARE-compliant clinical timeline of the patient's disease course.
