# Supplementary material for: Case Report: REM sleep without atonia in an adult with pediatric acute-onset neuropsychiatric syndrome: a case study and mechanistic insights
Source: Front Sleep. 2025 Nov 19;4:1654119. doi: 10.3389/frsle.2025.1654119 (PMC12713858; doi:10.3389/frsle.2025.1654119)
Supplement: Supplementary Table S1 — Multiple Sleep Latency Test (MSLT) findings at baseline assessment. [file Table_1.docx]

**Supplement[1]**

**Table S1. Multiple Sleep Latency Test (MSLT) findings at baseline assessment.**

|  | **Nap 1** | **Nap 2** | **Nap 3** | **Nap 4** | **Average** |
| --- | --- | --- | --- | --- | --- |
| **Lights Off** | 08:31:44 AM | 10:29:43 AM | 12:32:44 PM | 02:30:44 PM | - |
| **Lights On** | 08:51:43 AM | 10:51:13 AM | 12:53:13 PM | 02:51:13 PM | - |
| **Time In Bed** | 20.0 | 21.5 | 20.5 | 20.5 | 20.6 |
| **Sleep Time** | - | 15.0 | - | - | 3.8 |
| **Sleep Efficiency** | 0.0% | 69.8% | 0.0% | 0.0% | 18.2% |
| **Sleep Onset** | - | 10:35:43 AM | - | - | - |
| **Sleep Latency** | 20.0 | 6.0 | 20.0 | 20.0 | 16.5 |
| **REM Onset** | - | 10:46:43 AM | - | - | - |
| **REM Sleep Onset**  **Latency** | - | 11.0 |  |  | 11.0 |

**Abbreviations:** **MSLT** = Multiple Sleep Latency Test; **REM** = rapid eye movement sleep**; SOREMP** = sleep-onset REM period

**Table S2. CARE-compliant clinical timeline of the patient’s disease course.**

| **Date/Period** | **Clinical Events** | **Investigations** | **Treatments** |
| --- | --- | --- | --- |
| **2020** | Initial illness during COVID infection (mother & grandmother hospitalised, grandfather deceased). Onset of neuropsychiatric symptoms: motor/vocal tics, paranoia, regression. | None at onset. | None initially. |
| **2021** | Exacerbation during school exams with significant neuropsychiatric regression. | Elevated ASO titre (>800 IU/mL). | Propranolol (low dose) for anxiety under presumed functional tic disorder. |
| **2022** | Recurrent episodes with paranoia, sensory hypersensitivity, cognitive regression. | MRI and EEG clear; ANA positive (1:160, speckled); ferritin borderline low. | Supportive management only. |
| **2023** | Formal diagnosis of PANDAS following streptococcal relapse  severe exacerbation post-pregnancy termination. | Streptococcal testing positive during relapses. | Co-amoxiclav antibiotics (TID ×14 days, then daily ×28 days);  CBT for anxiety/emotional regulation. |
| **2024** | Baseline period with persistent sleep disturbance, cognitive complaints. | Overnight vPSG and MSLT: RSWA confirmed; AHI 0.1; sleep efficiency 90.8%.  MSLT: mean latency 16.5 min; 1 nap with REM latency 11 min; <2 SOREMPs. | None at time of vPSG (no antidepressants, no immunotherapy). |

A structured chronological overview of the patient’s clinical history according to CARE reporting standards. Key events include infectious triggers (COVID-19 in 2020, streptococcal relapses), symptom flare ups, laboratory investigations (elevated ASO titres, ANA positivity, ferritin), neuroimaging and EEG findings, treatments (antibiotics, propranolol, CBT), and sleep evaluations (vPSG and MSLT). The vPSG confirmed RSWA with negligible AHI (0.1 events/h), while the MSLT demonstrated normal mean latency (16.5 minutes) and 1 SOREMP. ***Abbreviations:*** **AHI** = apnoea–hypopnoea index; **ASO** = anti-streptolysin O; **ANA** = antinuclear antibody; **CBT** = cognitive behavioural therapy; **MSLT** = multiple sleep latency test; v**PSG** = video-polysomnography; **RSWA** = REM sleep without atonia; **SOREMPs** = sleep-onset REM periods.

[1] R. Ferri, F. Rundo, M. Manconi, G. Plazzi, O. Bruni, A. Oldani, L. Ferini-Strambi, and M. Zucconi, Improved computation of the atonia index in normal controls and patients with REM sleep behavior disorder. Sleep medicine 11 (2010) 947-9.
